# An open-source, automated machine learning approach for large-scale image retrieval for thoracic aorta analysis studies

**DOI:** 10.1093/jamiaopen/ooaf066

**Published:** 2025-07-14

**Authors:** Brian C Ayers, Aaron D Aguirre, Thoralf M Sundt, Michael T Lu, Arminder Jassar

**Affiliations:** Division of Cardiac Surgery, Massachusetts General Hospital, Boston, MA 02114, United States; Cardiology Division, Massachusetts General Hospital, Boston, MA 02114, United States; Wellman Center, Massachusetts General Hospital, Boston, MA 02114, United States; Division of Cardiac Surgery, Massachusetts General Hospital, Boston, MA 02114, United States; Department of Radiology, Cardiovascular Imaging Research Center, Massachusetts General Hospital & Harvard Medical School, Boston, MA 02114, United States; Division of Cardiac Surgery, Massachusetts General Hospital, Boston, MA 02114, United States

**Keywords:** convolutional neural network (CNN), machine learning, open-source, content-based image retrieval

## Abstract

**Objective:**

To develop an image retrieval pipeline capable of identifying specific series of thoracic aortic computed tomography (CT) scans from a diverse database.

**Materials and Methods:**

An automated image analysis pipeline was developed to select series that show the entire thoracic aorta with arterial phase contrast from within a heterogeneous institutional cohort of 4184 CT scans of the chest.

**Results:**

The automated pipeline identified 3435 (82%) studies from within the database that met criteria. Manual review confirmed 99.1% of the selected scans were accurately selected, and 93.6% of excluded scans were appropriately excluded.

**Discussion and Conclusion:**

We present an open-source, image-retrieval pipeline that, with a high degree of accuracy, can identify aortic imaging studies that meet specific criteria from within a heterogeneous collection of images. This pipeline serves as a framework that can be easily modified for other clinical use cases and can be deployed across multiple centers to promote multi-institutional research.

## Objective

Personalized risk prediction models could have significant impact on morbidity and mortality of patients with thoracic aortic aneurysms (TAAs). We aim to describe an automated approach to coalesce appropriate aortic images from a heterogeneous, large-scale database to create the data necessary to develop personalized risk models to help prevent life threatening aortic complications.^1^

## Background

Rupture or dissection of TAAs is a life-threatening event and results in significant patient morbidity even when treated. Moreover, the optimal timing of surgical intervention on TAA in asymptomatic patients in order to prevent these complications is poorly understood.^2^ EACTS/STS cardiothoracic surgical society guidelines are based predominately on maximum aneurysm diameter size and growth rate cutoffs but are neither sensitive nor specific.^3–5^

There is significant opportunity to apply modern machine learning techniques to develop a personalized aorta risk score based on patient specific anatomy.^6^ However, such an endeavor will require a large imaging dataset for model training. Furthermore, TAAs are relatively uncommon in the general population with an incidence of approximately 10 per 100 000 patient years,^7^ making it difficult to accrue the necessary number of patients at a single institution alone. Therefore, multi-institutional collaboration will be required to create an adequate dataset.

Collating multi-institutional imaging studies pose several challenges, most notably in the identification at scale of appropriate imaging studies across a number of different imaging platforms, hardware, and protocols.^8^ The DICOM standard was introduced decades ago for the attempted standardization of imaging metadata to assist with this heterogeneity problem.^9^ However, the flexibility and optionality associated with many DICOM variables have been shown to make the presence of a given variable highly inconsistent in large scale databases, greatly inhibiting the utility of employing DICOM data to solve this conundrum.^10–13^

Content-based image retrieval (CBIR) is an alternative method that relies on analyzing the content of the images themselves for creation of large-scale database of medical image studies.^14^ However, many of these techniques rely on the development of case specific machine learning models to adequately identify appropriate images.^11^^,^^15^ This approach is time and resource intensive, and most often results in specialized algorithms that are difficult to share or apply to other imaging projects.

In this study, we aimed to develop an algorithm that uses only open-source machine learning components to identify thin-cut (≤3 mm) series that show the entire thoracic aorta with arterial phase contrast from within a large-scale, heterogeneous institutional cohort of CT scans of the chest. The goal is for this algorithm to be easily deployed across multiple institutions, as well as to serve as an example framework for the development of similar CBIR techniques using open-source models that can be adapted to other medical image database studies.

## Methods

### Data collection

This was a retrospective study approved by our institutional review board (#2024P001266). To create the imaging dataset of patients with aortic pathology, a query was conducted of our institutional Research Patient Data Registry (RPDR) to identify patients that have CT imaging of the chest (identified by study code, Appendix Table SA) and a diagnosis of aortic aneurysm or dissection. RPDR is a centralized clinical data registry/warehouse created and maintained by the institution that incorporates all institutional clinical and billing data. The query included imaging studies from 2006 through 2020 that were obtained at any of 4 different hospitals within our health care system. A subset of these studies was subsequently extracted from the picture archiving and communication system (PACS) to be used for pipeline development and testing.

### Automated imaging review and analysis pipeline

All files included in the dataset were processed with an automated script (Python, v3.10.12) for image analysis that uses the following workflow:

1. *Select only axial orientation 3D CT image files.* Corrupted files were discarded. Axial orientation was determined using DICOM tags.
2. *Perform segmentation of the lungs, aorta, and brachiocephalic artery.* The open-source TotalSegmentator^16^^,^^17^ convolutional neural network package was used for segmentation without additional training or fine-tuning. The model was run in 3 mm fast mode to save on time and computational resource requirements. The model was run on a local GPU with 24GB memory (GeForce RTX 4090, NVIDIA, Santa Clara, CA, United States).
3. *Drop any series that does not contain any of the above segmentation labels*. This ensures that the series shows the chest (requires lungs to be visible) and shows the top of the aortic arch (requires brachiocephalic to be visible).
4. *Crop the CT scan to the thoracic cavity*. The previously obtained lung segmentation was used to identify the extent of the thoracic cavity.
5. *Calculate the median Hounsfield units (HU) of the aorta*. Voxel based radiomic calculations were conducted using an open-source package (Pyradiomics, v3.0.1).
6. *Keep only series with median HU in the aorta >150.* Excludes scans without sufficient arterial phase contrast. The cutoff threshold of 150 HU was chosen from a distribution representative of aortic contrast in the studies. All series with HU >150 were kept at this step. The final step 9 ensures only one series from each study was selected.
7. *Ensure the series shows the entire aorta.* This was achieved by dropping series where the previously aorta segmentation label touched the edge of the image either cranially or laterally, suggesting part of the aorta was cutoff.
8. *Drop if slice thickness is >3 mm.* Requirement to ensure adequate image resolution for future model development.
9. *Keep the series with the highest aortic median HU within each study.* For studies with multiple series remaining, the series with the highest median HU was selected for final inclusion. The end result of the pipeline is the series with maximum contrast in the aorta for each study that adequately displays the entire thoracic aorta.

### Pipeline performance

To determine the accuracy of each step of the automated pipeline in excluding studies, the studies excluded at each step were manually reviewed by practicing physicians to identify whether each exclusion was appropriate. Exclusion accuracy was defined as the number of series that were appropriately excluded divided by the total number of excluded studies manually reviewed for that step. To determine the performance of the algorithm in creating a final cohort of series that did in fact depict the entire thoracic aorta (inclusion accuracy), a 3D rendering of the aorta segmentation for all selected series was manually reviewed to assess whether the entire thoracic aorta was present. Inclusion accuracy was defined as the percent of series showing the entire thoracic aorta from the total number of series selected by the automated pipeline as meeting inclusion criteria.

## Results

Overall, the image database consisted initially of 2336 unique patients with a total of 4184 studies, consisting of 69 029 different series. There was significant diversity in the type of CT scanner, acquisition protocol used, and availability of contrast related metadata throughout the database (Table 1). In terms of DICOM tags, 85% of studies had some indication of contrast agent in at least one series, but <70% had start and end times present for the contrast to indicate timing which would be necessary for DICOM based series selection. Most of the studies were 512 × 512 pixels, with 649 ± 302 slices per study. This results in median [IQR] voxel resolution for the included cohort of 1.5 × 10^8^ [1.1 × 10^8^, 2.2 × 10^8^] voxels.

**Table 1.** Diversity in the model of CT scanner, acquisition protocol used, and availability of contrast related metadata.

Variable	Number of studies, no. (%)
**Manufacturer—17 unique values**	
Siemens	2691 (64%)
GE	582 (14%)
Bayer	345 (8%)
Other	566 (14%)
**Study description—132 unique values**	
CT angio chest with and without contrast	1215 (29%)
CT angio chest wwo contrast	672 (16%)
CT angio chest	563 (13%)
CT angio chest w contrast	358 (9%)
Cardiac^gated_thoracic_aorta_cta (adult)	162 (4%)
Other	1210 (29%)
**DICOM tag present**	
Contrast/Bolus Agent	3556 (85%)
Contrast/Bolus Start Time	2584 (62%)
Contrast/Bolus Stop Time	2759 (66%)

**Figure 1.**
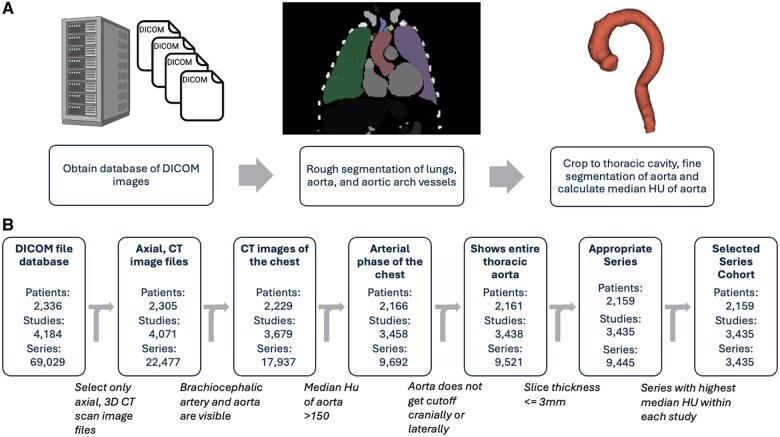
(A) Automated process for identifying appropriate series within each study. (B) Number of studies remaining after each exclusion step.

**Figure 2.**
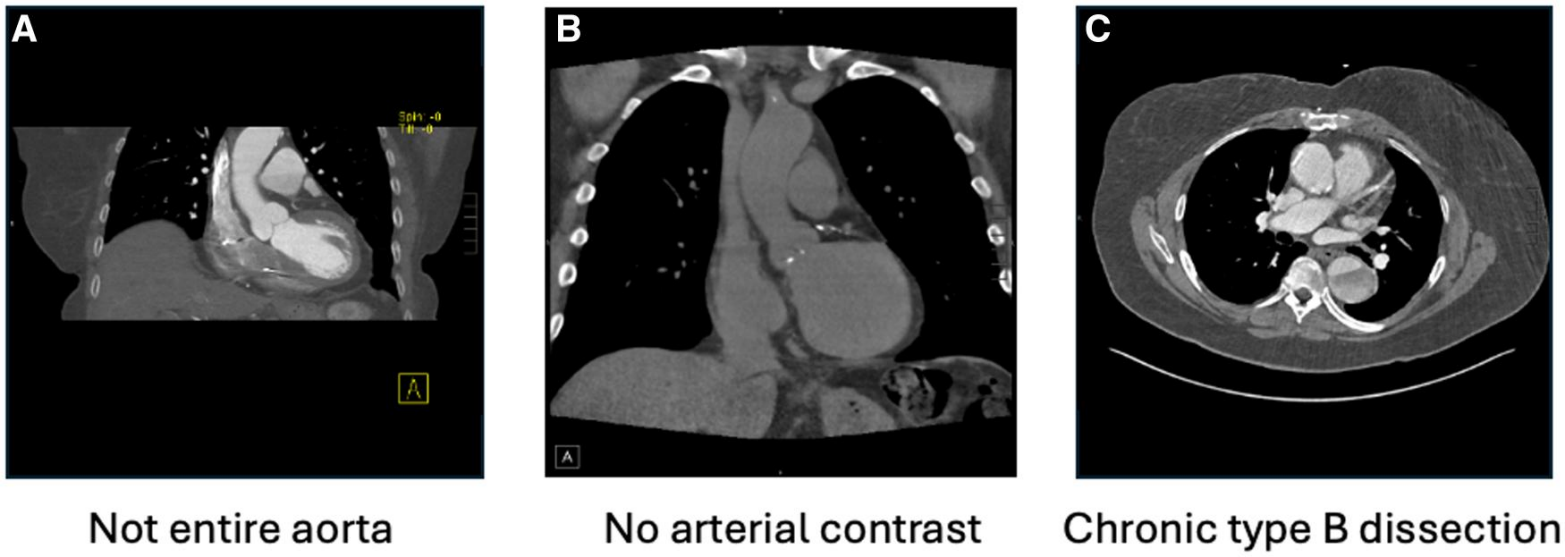
Representative examples of excluded cases for (A) not visualizing the entire thoracic aorta, (B) not being arterial phase contrast, and (C) for HU <150 caused by chronic type B dissection with intraluminal thrombus.

Figure 1 depicts the pipeline steps and how many patients, studies and series were excluded at each step, respectively. We manually reviewed each of the excluded studies to determine the accuracy of each exclusion step (Table 2). Each step of the pipeline had >90% exclusion accuracy in appropriately excluding the studies, except for the step dropping series with median HU <150 in the aorta which had only 80% accuracy. Of note, 55% of the incorrectly excluded studies in this step had thrombosed dissections in the thoracic aorta. As expected, there were many non-contrast series in the dataset with 45% of all series having a median HU <150 (Figure S1). However, after considering only the maximum series within a given study, only 6% of the studies failed to have at least one series with median HU >150. The overall median [interquartile range] HU at the study level across the cohort was 320 [262 399] HU following a non-normal distribution (Figure S2). Figure 2 demonstrates representative cases of studies that were appropriately and inappropriately excluded by the automated pipeline, including those without the entire thoracic aorta visible, without arterial phase contrast or with a chronic type B dissection with luminal thrombus.

**Table 2.** Manual review to assess the accuracy of each automated exclusion step.

	Studies excluded		Appropriately excluded	
Automated exclusion step	No.	%	No.	%
Does not contain axial, 3D CT image files	113	2.70%	110	97%
Does not have brachiocephalic artery and aorta visible	392	9.37%	392	100%
Aorta median Hounsfield units (HU) <150	221	5.28%	177	80%^a^
Aorta is cutoff cranially or laterally	20	0.48%	19	95%
Slice thickness ⩽3 mm	3	0.07%	3	100%

a 24/44 (55%) of incorrectly excluded studies depicted thrombosed aortic dissection.

**Figure 3.**
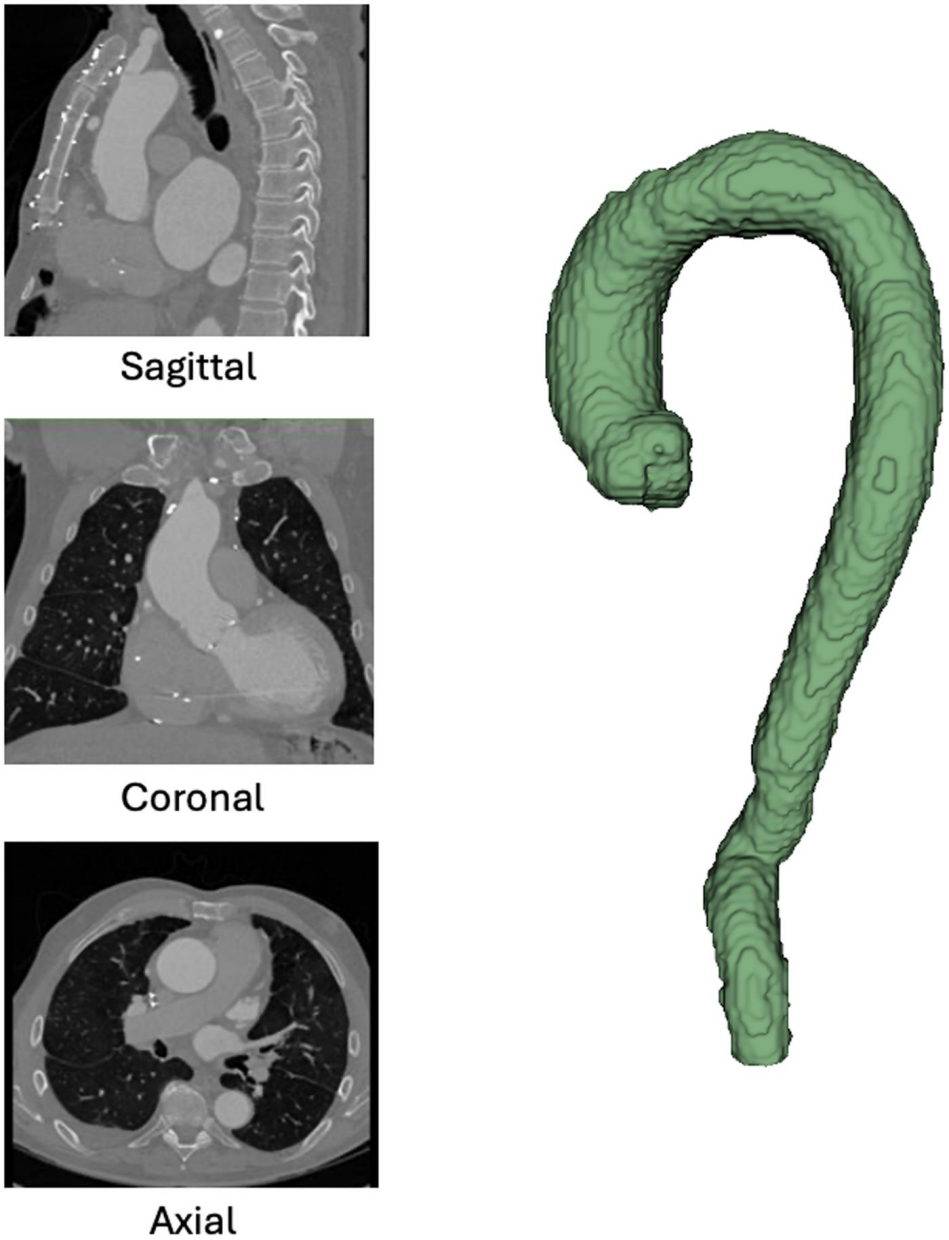
Example of an appropriately selected arterial phase study with the thoracic aorta autonomously segmented, cropped, and rendered into 3D for subsequent analysis.

In terms of inclusion accuracy, manual review of 3D renderings of the aorta segmentations for each of the 3435 selected series found that 3406 (99.1%) series were appropriately identified as depicting the entire thoracic aorta with sufficient IV contrast to perform segmentation. Figure 3 demonstrates an example of an appropriately selected arterial phase series with the thoracic aorta autonomously segmented and cropped for subsequent analysis.

## Discussion

The combination of an explosion in the vast quantity of medical imaging being obtained clinically,^18^ and advances in machine learning image analysis techniques^19^ creates a prime opportunity to create personalized, image based prognostic models for clinically significant questions.^6^^,^^8^ However, to make use of these data and techniques, automated approaches for the identification of appropriate series at scale are needed. To this end, we developed an automated image selection pipeline that shows a high degree of accuracy in autonomously identifying series within a diverse dataset that depict the entire thoracic aorta with arterial phase contrast—the first step towards creating a personalized, prognostic image-based risk model for aortic pathology.

There are number of important potential advantages of the presented pipeline compared to other previously reported image retrieval techniques.^10^^,^^12^^,^^14^^,^^15^^,^^20^ First, it is developed using entirely open-source tools. This allows for broad dissemination and application within any academic or commercial projects. Second, it does not require any task specific training of specialized machine learning algorithms. This decreases the computational requirements during development and makes it more easily adaptable to new use cases. The open-source segmentation convolutional neural network employed in the pipeline has 117 different segmentation classes for computed tomography (CT) imaging.^16^ While this study focuses on identifying the thoracic aorta, the pipeline could be easily modified and immediately implemented for any number of other use cases without any additional model training required. And lastly, it utilizes minimal DICOM tag data (only the orientation tag and slice thickness), thereby reducing the risk of the pipeline breaking if insufficient or different DICOM data is included across multi-institutional studies. Despite relying predominately on analyzing the image files directly, a task which can be computational expensive, the open-source tools chosen are computationally small enough to be run locally with only modest hardware requirements, allowing sensitive patient data to remain securely behind institutional firewalls.

The pipeline performed well, as 99.1% of the selected series did appropriately depict the thoracic aorta, and 93.6% of the excluded scans were accurately excluded. The majority of the incorrectly excluded studies were dropped because of low median HU in the aorta. Manual review showed that these studies did have arterial phase contrast, but the majority also had a thrombosed chronic dissection that thereby decreased the median HU below the cutoff threshold. The threshold of 150 HU was chosen after examining the distribution of median HU in a subset of aortic contrast and non-contrast studies and could easily be shifted to include more of these scans. While these inappropriately excluded studies still represent only a small percent of the total cohort, it highlights the need for quality checks of each step of any automated image retrieval pipeline and the need for potential additional steps or modifications for a given use case. If these thrombosed chronic dissection images were crucial to be included in the final dataset, pipeline modifications could be explored such as using a different HU cutoff value, a different radiomics metric rather than median HU, or a separate specialized pathway for this subset of scans.

Limitations of this study include it being a single healthcare system, retrospective study of a cohort of patients with specific aortic diagnoses. Any implementation or modification of the presented pipeline should include local testing to ensure continued accuracy.

## Conclusion

In conclusion, we present an open-source, image-retrieval pipeline that can accurately identify arterial phase series that depict the entire thoracic aorta from a heterogeneous cohort of thoracic CT scans. The present pipeline can be immediately applied to create multi-institutional databases of aortic imaging, as well as easily modified for other clinical use cases.

## Supplementary Material

ooaf066_Supplementary_Data

## Data Availability

The image files are unable to be released due to patient privacy. The software code will be made open-source and available on GitHub at the time of publication.
