# Supplementary material for: An open-source, automated machine learning approach for large-scale image retrieval for thoracic aorta analysis studies
Source: JAMIA Open. 2025 Jul 14;8(4):ooaf066. doi: 10.1093/jamiaopen/ooaf066 (PMC12257624; doi:10.1093/jamiaopen/ooaf066)
Supplement: ooaf066_Supplementary_Data [file ooaf066_supplementary_data.zip › Appendix.docx]

**APPENDIX**

**Table A. Institutional database query to identify radiographic studies that represent computed tomography (CT) imaging of the chest.**

| **Study Name** |
| --- |
| CTA CHEST (Test:BC193) |
| CTA CHEST (Test:CT093) |
| CTA CHEST (Test:ECT093) |
| CTA Chest W&W/O CON (Test:CTACOC) |
| CTA Chest W&W/O CON (Test:CTANGC) |
| CTAChest w/Con (Test:CTACOR) |
| CT CARDIAC (Test:CT.CA.HEART) |
| CT CARDIAC (Test:CT.CA.HEART) |
| CT CARDIAC GATED (Test:CT.CA.HEARTGA) |
| CT CARDIAC VEINS WITH AND WITHOUT CONTRAST (Test:CT.CA.HEARTVN@) |
| CT CARDIAC WITH AND WITHOUT CONTRAST(NOT FOR CORONARY ASSESSMENT) (Test:CT.CA.HEART@) |
| CT CARDIAC WITH CONTRAST (Test:CT.CA.HEART+) |
| CT ANGIO CHEST (Test:CT.VA.CHESTAG) |
| CT ANGIO CHEST (Test:CT.VA.CHESTAG) |
| CT ANGIO CHEST WITH AND WITHOUT CONTRAST (Test:CT.VA.CHESTAG@) |
| CT ANGIO CHEST WITH CONTRAST (Test:CT.VA.CHESTAG+) |
| CT ANGIO CHEST (Test:CT.VA.CHESTAG) |
| CT ANGIO CHEST (Test:CT.VA.CHESTAG) |
| CT ANGIO CHEST WITH AND WITHOUT CONTRAST (Test:CT.VA.CHESTAG@) |
| CT ANGIO CHEST WITH CONTRAST (Test:CT.VA.CHESTAG+) |
| CT ANGIO CORONARY ARTERIES WITH AND WITHOUT CONTRAST (Test:CT.VA.HEART@) |
| CTA for Gated CAP (Test:CTANGVP) |
| CTChest WWO-Vasc (Test:CTCHVAWWO) |
| CTChestW-Vasc (Test:CTCHVAW) |
| CTA CHEST (Test:BC193) |
| CTA CHEST (Test:CT093) |
| CTA CHEST (Test:ECT093) |
| CTA Chest W&W/O CON (Test:CTACOC) |
| CTA Chest W&W/O CON (Test:CTANGC) |
| CTAChest w/Con (Test:CTACOR) |
