# Supplementary material for: An open-source, automated machine learning approach for large-scale image retrieval for thoracic aorta analysis studies
Source: JAMIA Open. 2025 Jul 14;8(4):ooaf066. doi: 10.1093/jamiaopen/ooaf066 (PMC12257624; doi:10.1093/jamiaopen/ooaf066)
Supplement: ooaf066_Supplementary_Data [file ooaf066_supplementary_data.zip › SuppFigures.docx]

**Supplementary Figures**


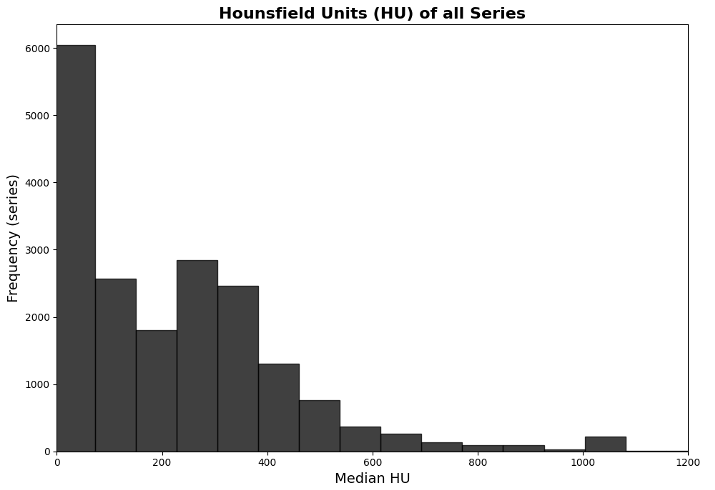
**Figure S1.** Median Hounsfield Units (HU) for each series within the database. Red dotted line represents the HU >150 threshold.


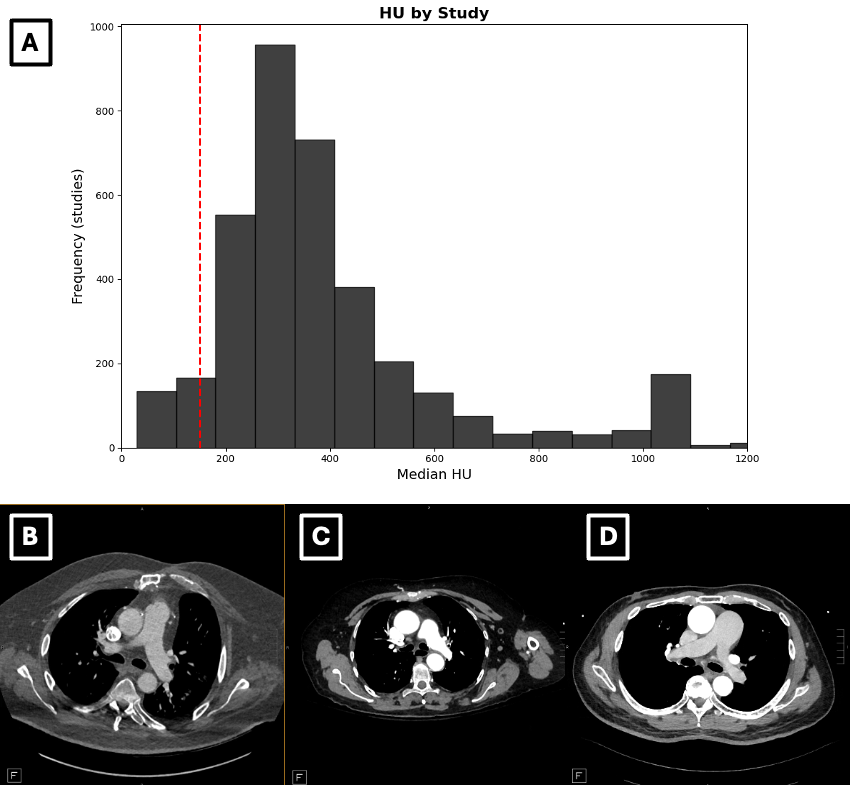


**Figure S2.** (A) The median Hounsfield Units (HU) of the maximum series within each study. Red dotted line represents the HU >150 threshold. Representative series with HU in the (B) first quartile, (C) median, and (D) third quartile.
